# Innovation in prenatal care: a global review of implementing group prenatal care and impacts on women, children, and health care providers

**DOI:** 10.3389/fgwh.2026.1910318

**Published:** 2026-09-25

**Authors:** Megan E. Austin-Cherry, Deborah L. Billings

**Affiliations:** 1 Department of Health Promotion, Education, and Behavior, University of South Carolina, Columbia, SC, United States; 2 Institute for Families in Society, University of South Carolina, Columbia, SC, United States; 3 Department of Population Health Leadership and Analytics, University of Texas at Tyler, Tyler, TX, United States; 4 Group Care Global, Philadelphia, PA, United States

**Keywords:** antenatal care, group care, implementation research, maternal and child health, prenatal care

## Abstract

**Background:**

Over the past 15 years, group-based prenatal care — bringing together 8 to 12 pregnant individuals for shared health assessment, interactive learning, and community building — has gained global interest as an alternative to traditional individual prenatal appointments.

**Objective:**

This mini-review synthesizes global research on the impact of group prenatal care on women, infants, and health care providers.

**Methods:**

A narrative approach was used to synthesize peer-reviewed literature of international group prenatal care models and their multiple impacts on women, children, and providers using predefined eligibility criteria and thematic synthesis.

**Results:**

Group prenatal care consistently improved women's empowerment, social support, mental health, health literacy, and care adherence. Women showed lower rates of gestational diabetes and pre-eclampsia. Infants benefited through improved maternal behaviors, including breastfeeding initiation and immunization. Providers reported stronger patient relationships, though challenges in facilitation, time management, and workload were noted.

**Conclusion:**

Group prenatal care offers clear benefits across diverse global settings. Successful implementation requires adequate resources, facilitation training, and stakeholder buy-in. Longitudinal research on long-term child health outcomes is needed.

## Introduction

Traditional prenatal care is focused on individual appointments between the pregnant person and health care provider. However, in the past 15 years, group-based prenatal care has been garnering interest and is being implemented around the world. The group-based model of prenatal care was developed in the 1990s by nurse-midwife Sharon Schindler Rising, known as CenteringPregnancy®, with its three core elements, namely clinical health assessment, interactive group-based learning, and community building (1). Group prenatal care brings together 8 to 12 pregnant persons of similar gestational age to learn about their bodies, their pregnancy, take leadership in their health care through learning how to take and report their own health vitals, socialize with people having a shared experience, and to receive a health assessment (2, 3). All groups are conducted in a circle, and most groups are facilitated by obstetric providers, especially certified nurse-midwives and nurse practitioners (4).

Rising originally designed the group model of prenatal care to address gaps identified in prenatal care, such as limited focused provider-patient time, lack of education about what to expect in pregnancy and birth, limited opportunities for women to socialize and gain support from others during a similar stage of pregnancy, and disparities in health outcomes for low-income women (3). Documented success in improved neonatal and maternal health outcomes in the United States, including reduced preterm birth, improved breastfeeding initiation and duration, and decreased cesarean delivery, raised interest in implementing the model more broadly across different contexts (2, 5).

Regardless of global location, a group model of prenatal care empowers women to have a role in their health, improve satisfaction with their prenatal care, and offer peer connection and support (6). In low-and middle-income countries (LMICs) specifically, the group prenatal care model is intended to overcome barriers to accessing pregnancy education and prenatal care by alleviating time constraints of one-on-one sessions and increasing trust in providers through group interactions (7).

A strong body of global research now exists to show the impact of group prenatal care on a range of health outcomes and the experiences of women, newborns, and providers in offering and receiving care. This mini-review summarizes that research, and Supplementary Table S1 outlines the articles, study location, and study details for all articles included in the review.

## Methods

This mini-review used a narrative review (8) approach to identify and synthesize data from articles with the intention to answer the following question: how does a group model of prenatal care in international settings around the world impact the health and experiences of women, children, and providers? Peer-reviewed original and review articles were identified from PubMed and Google Scholar with key words of “centering pregnancy”, “group prenatal care”, “group postnatal care”, and “group care”. Titles and abstracts were screened for relevance to the review objectives, followed by assessing the full text of potentially eligible articles. Articles were included if they were published in English, the research was conducted outside of the United States, and the research focused on impacts of a group model of care for pregnant and parenting women or the session providers as required by the scope of this review. A date range was not specified as analyzing a group prenatal model of care is a newer area of research, especially outside of the United States.

Since this study was conducted as a narrative mini-review rather than a formal systematic review, quantitative measures of the study selection process, including the number of records screened and excluded at each stage, were not prospectively documented. Nevertheless, predefined eligibility criteria and thematic relevance guided the identification and selection of studies to promote methodological transparency and ensure comprehensive coverage of the relevant literature. All selected articles were synthesized under three main themes: perspectives of and health impacts for women participating in group sessions, health impacts for the children of women participating in group sessions, and perspectives of and impacts for providers implementing group sessions.

## Impact of group care model of prenatal care on women

The impact of a group model of prenatal care for women greatly aligns with the tenants on which the model was built. The group model of prenatal care consistently increases women's sense of empowerment (9–19) and social support (9, 10, 12, 18–22). Empowerment is often connected with having greater participation in one's own health, greater engagement with health care providers as well as other pregnant people, and the space to ask questions and be heard (23). Social support is often tied to improved mental health. The mental health impact of group prenatal care has been repeatedly recorded through observing an increase in happiness and subsequent decrease in depressive symptoms during pregnancy (24), lower rates of post-partum depression (25), and decreased stress and concern for labor and delivery (26, 27).

In addition to the mental health implications of group prenatal care, women who attended group sessions were more likely to return and consistently receive prenatal care throughout their pregnancy (9, 26, 28–30), utilize other health care resources (31), and deliver in a hospital (29). Group sessions also promoted a more equal and improved relationship between women and their health care provider, which contributed to feelings of empowerment (19, 20). Through these improved relationships and regular prenatal care sessions, women experiencing group care have greater levels of knowledge about birthing, the post-partum period, and parenting when compared to women who received individualized prenatal care (10, 11, 20, 27, 31–33). This improved knowledge was especially prominent among vulnerable and high-risk populations, such as adolescent and low-income mothers. Through increased knowledge and repeated exposure to health messaging in sessions, women attending group prenatal care also had improved health literacy (34), and were more aware of danger signs during pregnancy (17, 18, 24, 35). Increased knowledge of danger signs led to higher healthcare utilization but also emphasized a need for more preventative health behaviors.

Women in group prenatal care were more likely to talk to their partners about reproductive health (36), use modern family planning methods post-partum (37), and put into practice better health behaviors overall (16, 26, 31, 38). These health behaviors included less alcohol consumption after birth, increased physical activity, and increased healthy eating. The increased knowledge, preventative health behaviors, and early action to warning signs had many positive physical health outcomes for mothers as well. Women in group prenatal care had lower prenatal weight gain (25) and were less likely to have pre-eclampsia (9) and gestational diabetes (39). While some studies found rates of cesarean sections remaining the same regardless of prenatal care delivery method (40), one study found lower rates of cesarean section for women in group care (25). Whether mental or physical, the evidence is clear that group prenatal care has a positive influence on the health of women during pregnancy as well as postpartum, regardless of location.

## Impact of group care model of prenatal care on infants

While most research has been focused on the impact on the health of pregnant women and postpartum mothers, some studies have also assessed the influence of group prenatal care on the infant. Outcomes range from maternal practices influencing child health to direct infant birth outcomes such as prematurity and low birth weight. A group model of prenatal care was seen to increase maternal practices influencing their child's health regardless of health behavior or location. Mothers with a known positive HIV status who received group prenatal care were more likely to take steps to prevent HIV transmission during birth (41) and get their infant tested for HIV after birth, although this latter finding was not statistically significant (42). Additionally, mothers who attended group prenatal care sessions were more likely to use insecticide treated nets over their infant's crib (43) and immunize their child (28, 44). Women who received group prenatal care also had higher intention to and initiation of breastfeeding with the goal to exclusively breastfeed (25, 39, 45, 46). Each of these behaviors fosters infant health after birth. Multiple studies found that mothers who attended group prenatal care sessions were more likely to know newborn danger signs and seek help if they noticed a concern (9, 47).

Results vary in terms of the influence of group prenatal care on infant birth outcomes in low- and middle-income countries. Some studies show that there is a lower risk of premature birth and low birth weight of the infant due to group care participation (45), while other studies show these health outcomes are comparable (25, 48). One study focused specifically on adolescent mothers found that the age of the mother had a larger influence on infant health than the delivery method of prenatal care (49).

Regardless of the maternal behaviors that influence their child's health, higher income countries specifically showed less of an overall difference in infant birth outcomes in relation to the way in which prenatal care was delivered but emphasized the importance of needing to study the impact of group prenatal care on providers (40).

## Impact of group care model of prenatal care on health care providers

While the benefits of a group model of prenatal care are seen for women and children, the impact on health care providers is varied, although overall is reported as being positive. Many providers expressed enjoying facilitating the group sessions, mentioning an opportunity to build stronger relationships and increase communication with their patients (10, 50, 51) while in a more comfortable setting than what is typical (50, 52). Some studies showed that their providers had increased personal and professional fulfillment (10, 51, 53) from leading group sessions. For many, this was due to a change of pace, or an opportunity to not just “clear the queue” but have more in-depth interactions with patients (10). This increased fulfillment was also a result of providers feeling that they were able to offer higher quality care to their patients because of the group care sessions (10, 54, 55). Specifically, increased quality of care was reflected when providers were able to still tailor the group care sessions to the individuals within the group, instead of only offering generic information. When tailoring occurred, providers were able to increase their cultural understanding of who they were serving (56).

Tailoring was one of the largest challenges for many providers in facilitating groups, though, as there may be a variety of women in the room who all have unique experiences and health literacy levels (50, 57). Other challenges to facilitation included time management (22) and other soft skills necessary for facilitation (58), such as managing when women offered incorrect advice to their peer (22), or group dynamics became challenging to manage (21). Additional facilitation challenges included limited physical space for the group (59), typical practice standards that providers were used to following not aligning with the group model and causing inner turmoil (22), and the inability to see as many individual patients with time taken by the group (59).

Overall, some providers found leading the groups to be taxing (22), and this extra responsibility did not lower their workload. At the same time, providers expressed happiness or excitement that women were able to find peer support and receive more education through the group model (50–53, 56).

## Limitations

While many studies have identified the influence of a group model of prenatal care on the health and experiences of mothers, children, and providers, this is the first review, to our knowledge, to summarize the impact across all categories. Despite this novel approach towards assessing the literature to date, we acknowledge the limitations of this mini-review. First, this is a mini-review, not a full systematic review or meta-analysis. While the articles were identified and included through a structured process, the review was not registered, so selected studies may be subject to selection bias and all eligible studies may not be captured. Second, this mini-review was focused solely on contexts outside of the United States, limiting the knowledge base from where much research has been conducted on a group model of care. Third, while all international settings were eligible to be included, much of the research was conducted in low- or –middle income settings. This may limit the generalizations of the findings from this review. Fourth, while most studies focused on health and experiences of women and children were based in rigorous methods, many of the studies focused on provider experiences were evaluating feasibility, acceptability, and perspectives through less rigorous methods (see Supplementary Table 1). Despite these limitations, this mini-review offers important insight into the impact of utilizing a group model of prenatal care.

## Conclusion

This mini-review outlines and highlights the impact of a group model of prenatal care with evidence generated throughout the world. Group prenatal care has a clear influence on the health of pregnant and postpartum women through empowerment, social support, mental, and physical health. The health care experience for women through group prenatal care is generally very positive and even transformative, leaving mothers with a broader social network, more information about their bodies and resources, greater trust in health care providers, and a stronger sense of their abilities to be engaged in their health decisions.

Through both maternal behaviors and infant birth outcomes, group prenatal care positively influences the health of developing pregnancy as well as the infant postpartum. Longitudinal research is needed to demonstrate the medium and long-term impact group prenatal care can have on children's health.

Widespread integration of a group prenatal care model would contribute to the urgent need to improve the health and outcomes of pregnant and postpartum people as well as their infants, around the world. For integration to be successful, health care systems need to consider the resources needed for implementation, including time and space for groups and facilitation training for health care providers to effectively facilitate groups. Buy-in by and satisfaction with group care by health care providers, administrators, patients, and the larger community are necessary for this systems transformation to be accepted, fostered, and ultimately demanded by these key stakeholders.

## References

[1] Medical Reports. Group Prenatal Care: Centering Pregnancy. Medical Update. (2014) (Accessed Jun 11, 2026). Available online at: https://medicalupdate.pennstatehealth.org/obstetrics-gynecology/group-prenatal-care-centering-pregnancy/

[2] Andrade-Romo Z Heredia-Pi IB Fuentes-Rivera E Alcalde-Rabanal J Cacho LBB Jurkiewicz L. Group prenatal care: effectiveness and challenges to implementation. Rev Saude Publica. (2019) 53:85. 10.11606/s1518-8787.2019053001303 31576945 PMC6763281

[3] Rising SS. Centering pregnancy: an interdisciplinary model of empowerment. J Nurse Midwifery. (1998) 43(1):46–54. 10.1016/S0091-2182(97)00117-1 9489291

[4] American College of Obstetricians and Gynecologists. Group Prenatal Care. (2018) (Accessed Jun 11, 2026). Available online at: https://www.acog.org/clinical/clinical-guidance/committee-opinion/articles/2018/03/group-prenatal-care

[5] Tilden EL Hersh SR Emeis CL Weinstein SR Caughey AB. Group prenatal care: review of outcomes and recommendations for model implementation. Obstet Gynecol Surv. (2014) 69(1):46. 10.1097/OGX.0000000000000025 25102251

[6] Novick G Sadler LS Kennedy HP Cohen SS Groce NE Knafl KA. Women’s experiences of group prenatal care. Qual Health Res. (2011) 21(1):97–116. 10.1177/1049732310378655 20693516 PMC3085399

[7] Gaur BPS Vasudevan J Pegu B. Group antenatal care: a paradigm shift to explore for positive impacts in resource-poor settings. J Prev Med Public Health. (2021) 54(1):81–4. 10.3961/jpmph.20.349 33618503 PMC7939754

[8] Sukhera J. Narrative reviews: flexible, rigorous, and practical. J Grad Med Educ. (2022) 14(4):414–7. 10.4300/JGME-D-22-00480.1 35991099 PMC9380636

[9] Casella JBM Julmisse M Adeyemo OO Vital Julmiste TM Illuzzi JL. Integrated group antenatal and pediatric care in Haiti: a comprehensive care accompaniment model. PLoS One. (2024) 19(7):e0300908. 10.1371/journal.pone.0300908 38995942 PMC11244772

[10] Grenier L Onguti B Whiting-Collins LJ Omanga E Suhowatsky S Winch PJ. Transforming women’s and providers’ experience of care for improved outcomes: a theory of change for group antenatal care in Kenya and Nigeria. PLoS One. (2022) 17(5):e0265174. 10.1371/journal.pone.0265174 35503773 PMC9064109

[11] Hunter LJ Da Motta G McCourt C Wiseman O Rayment JL Haora P. Better together: a qualitative exploration of women’s perceptions and experiences of group antenatal care. Women Birth. (2019) 32(4):336–45. 10.1016/j.wombi.2018.09.001 30253938

[12] Dai J Yu Y Wang Y Huang Y Liu J Lin Y. The experience of pregnant women in the health management model of internet-based centering pregnancy: a qualitative study. Int J Women’s Health. (2022) 14:1281–9. 10.2147/ijwh.s375675 PMC9464633 36105790

[13] Ghani RMA. Effect of group centering pregnancy on empowering women with gestational hypertension. J Health Med Nu. (2018) 55.

[14] Malchi F Abedi P Iravani M Maraghi E Mohammadi E Saadati N. The effect of group prenatal care on empowerment of pregnant adolescents: a randomized controlled trial. Sultan Qaboos Univ Med J. (2023) 23(4):502–9. 10.18295/squmj.6.2023.041 38090240 PMC10712397

[15] Patil CL Klima CS Leshabari SC Steffen AD Pauls H McGown M. Randomized controlled pilot of a group antenatal care model and the sociodemographic factors associated with pregnancy-related empowerment in Sub-Saharan Africa. BMC Pregnancy Childbirth. (2017) 17(Suppl 2):336. 10.1186/s12884-017-1493-3 29143624 PMC5688418

[16] Sayed HAE; Ae EM. Effect of centering pregnancy model implementation on prenatal health behaviors and pregnancy related empowerment. Am J Sci. (2018) 7(6):314–24.

[17] Somji A Ramsey K Dryer S Makokha F Ambasa C Aryeh B. Taking care of your pregnancy”: a mixed-methods study of group antenatal care in Kakamega county, Kenya. BMC Health Serv Res. (2022) 22:969. 10.1186/s12913-022-08200-1 35906642 PMC9336107

[18] Thapa P Bangura AH Nirola I Citrin D Belbase B Bogati B. The power of peers: an effectiveness evaluation of a cluster-controlled trial of group antenatal care in rural Nepal. Reprod Health. (2019) 16(1):150. 10.1186/s12978-019-0820-8 31640770 PMC6805428

[19] Horn A Leister N Lazar J Bradley S McCourt C. Women’s experiences of group antenatal care: findings from a systematic review and feminist qualitative meta-synthesis. BMC Pregnancy Childbirth. (2026). 10.1186/s12884-026-09503-5

[20] Musabyimana A Lundeen T Butrick E Sayinzoga F Rwabufigiri BN Walker D. Before and after implementation of group antenatal care in Rwanda: a qualitative study of women’s experiences. Reprod Health. (2019) 16(1):90. 10.1186/s12978-019-0750-5 31248425 PMC6595554

[21] Craswell A Kearney L Reed R. “Expecting and connecting” group pregnancy care: evaluation of a collaborative clinic. Women Birth. (2016) 29(5):416–22. 10.1016/j.wombi.2016.03.002 27038560

[22] McNeil DA Johnston JC. Implementing CenteringParenting in well child clinics: mothers’ nurses’ and decision makers’ perspectives. J Community Public Nurs. (2016) 2(3):134. 10.4172/2471-9846.1000134

[23] Horn A Orgill M Billings DL Slemming W Van Damme A Crone M. Belonging: a meta-theme analysis of women’s community-making in group antenatal and postnatal care. Front Public Health. (2025) 13:1506956. 10.3389/fpubh.2025.1506956 40078777 PMC11897044

[24] Ahrne M Byrskog U Essén B Andersson E Small R Schytt E. Group antenatal care compared with standard antenatal care for Somali- Swedish women: a historically controlled evaluation of the hooyo project. BMJ Open. (2023) 13(1):e066000. 10.1136/bmjopen-2022-066000 36697050 PMC9884917

[25] Yang X Li L Zhou R Xia J Li M Zhang C. Effects of the online and offline hybrid continuous group care on maternal and infant health: a randomized controlled trial. BMC Pregnancy Childbirth. (2023) 23(1):629. 10.1186/s12884-023-05882-1 37658338 PMC10472587

[26] Marzouk T Abd-Allah IM Shalaby H. Effect of applying centering pregnancy model versus individual prenatal care on certain prenatal care outcomes. Clin Nurs Stud. (2018) 6(2)91–100. 10.5430/cns.v6n2p91

[27] Swift EM Zoega H Stoll K Avery M Gottfreethsdottir H. Enhanced antenatal care: combining one-to-one and group antenatal care models to increase childbirth education and address childbirth fear. Women Birth. (2020) 34:381–8. 10.1016/j.wombi.2020.06.008 32718800

[28] Eluwa GI Adebajo SB Torpey K Shittu O Abdu-Aguye S Pearlman D. The effects of centering pregnancy on maternal and fetal outcomes in northern Nigeria; a prospective cohort analysis. BMC Pregnancy Childbirth. (2018) 18(1):158. 10.1186/s12884-018-1805-2 29751797 PMC5948874

[29] Grenier L Suhowatsky S Kabue MM Noguchi LM Mohan D Karnad SR. Impact of group antenatal care (G-ANC) versus individual antenatal care (ANC) on quality of care, ANC attendance and facility-based delivery: a pragmatic cluster-randomized controlled trial in Kenya and Nigeria. PLoS One. (2019) 14(10):e0222177. 10.1371/journal.pone.0222177 31577797 PMC6774470

[30] Patil CL Klima CS Steffen AD Leshabari SC Pauls H Norr KF. Implementation challenges and outcomes of a randomized controlled pilot study of a group prenatal care model in Malawi and Tanzania. Int J Gynaecol Obstet. (2017) 139(3):290–6. 10.1002/ijgo.12324 28905377 PMC5673548

[31] Wagijo M Crone MR Bruinsma-van Zwicht B Van Lith JM Billings DL Rijnders ME. Contributions of CenteringPregnancy to women’s health behaviours, health literacy, and health care use in The Netherlands effect of CenteringPregnancy on women’s health behaviours, health literacy and healthcare use. Prev Med Rep. (2023). 10.1016/j.pmedr.2023.102244 37415970 PMC10320596

[32] Hetherington E Tough S McNeil D Bayrampour H Metcalfe A. Vulnerable women’s perceptions of individual versus group prenatal care: results of a cross-sectional survey. Matern Child Health J. (2018) 22(11):1632–8. 10.1007/s10995-018-2559-1 29934926

[33] Ratzon R Cohen A Hadar A Froimovici M Bilenko N. Impact of group vs. Individual prenatal care provision on women’s knowledge of pregnancy-related topics: an open, controlled, semi-randomized community trial. J Clin Med. (2022) 11:5015. 10.3390/jcm11175015 36078944 PMC9457062

[34] Lori JR Ofosu-Darkwah H Boyd CJ Banerjee T Adanu RMK. Improving health literacy through group antenatal care: a prospective cohort study. BMC Pregnancy Childbirth. (2017) 17(1):228. 10.1186/s12884-017-1414-5 28705179 PMC5513199

[35] Adaji SE Jimoh A Bawa U Ibrahim HI Olorukooba AA Adelaiye H. Women's experience with group prenatal care in a rural community in northern Nigeria. Int J Gynaecol Obstet. (2019) 145(2):164–9. 10.1002/ijgo.12788 30779108 PMC6449218

[36] Jeremiah RD Patel DR Chirwa E Kapito E Mei X McCreary LL. A randomized group antenatal care pilot showed increased partner communication and partner HIV testing during pregnancy in Malawi and Tanzania. BMC Pregnancy Childbirth. (2021) 21:790. 10.1186/s12884-021-04267-6 34819018 PMC8611988

[37] Lori JR Chuey M Munro-Kramer ML Ofosu-Darkwah H Adanu RMK. Increasing postpartum family planning uptake through group antenatal care: a longitudinal prospective cohort design. Reprod Health. (2018) 15(1):208. 10.1186/s12978-018-0644-y 30558677 PMC6296041

[38] El Aliem RSA Mustafa SA Ellatef MABA. Effect of applying pregnancy centered care model on pregnant women health behaviors and prenatal controls. Am J Nurs Res. (2019) 7(2):219–27.

[39] Hawley NL Faasalele-Savusa K Faiai M' Suiaunoa-Scanlan L Loia M Ickovics JR. A group prenatal care intervention reduces gestational weight gain and gestational diabetes in American Samoan women. Obesity. (2024) 32(10):1833–43. 10.1002/oby.24102 39256170 PMC11755376

[40] Hodgson ZG Saxell L Christians JK. An evaluation of interprofessional group antenatal care: a prospective comparative study. BMC Pregnancy Childbirth. (2017) 17(1):297. 10.1186/s12884-017-1485-3 28882131 PMC5590183

[41] Akunzirwe R Bakeera-Kitaka S Kalyango JN Zalwango JF Ssemasaazi JA Okello T. Optimal utilization of prevention of mother-to-child transmission of HIV services among adolescents under group versus focused antenatal care in eastern Uganda. PLoS One. (2022) 17(11):e0275905. 10.1371/journal.pone.0275905 36318523 PMC9624396

[42] Teasdale CA Odondi J Kidiga C Choy M Fayorsey R Ngeno B. Group antenatal care for improving retention of adolescent and young pregnant women living with HIV in Kenya. BMC Pregnancy Childbirth. (2022) 22(1):208. 10.1186/s12884-022-04527-z 35291978 PMC8925235

[43] Noguchi L Grenier L Kabue M Ugwa E Oyetunji J Suhowatsky S. Effect of group versus individual antenatal care on uptake of intermittent prophylactic treatment of malaria in pregnancy and related malaria outcomes in Nigeria and Kenya: analysis of data from a pragmatic cluster randomized trial. Malar J. (2020) 19(1):51. 10.1186/s12936-020-3099-x 31996209 PMC6990503

[44] Johnston JC McNeil D Van Der Lee G MacLeod C Uyanwune Y Hill K. Piloting CenteringParenting in two Alberta public health well-child clinics. Public Health Nurs. (2017) 34(3):229–37. 10.1111/phn.12287 27501111

[45] Nicolau P Rodriguez S Maso P Agramunt S Garcia E Sala A. Impact of group prenatal care support on breastfeeding initiation rates and other maternal and perinatal outcomes. Obstet Gynecol Res. (2018) 1(2):38–44. 10.26502/ogr.4560007

[46] Maldonado LY Songok JJ Snelgrove JW Ochieng CB Chelagat S Ikemeri JE. Promoting positive maternal, newborn, and child health behaviors through a group-based health education and microfinance program: a prospective matched cohort study in western Kenya. BMC Pregnancy Childbirth. (2020) 20(1):288. 10.1186/s12884-020-02978-w 32398156 PMC7216653

[47] Apetorgbor V Awini E Ghosh B Zielinski R Amankwah G Kukula VA. The impact of group antenatal care on newborns: results of a cluster randomized control trial in eastern region, Ghana. BMC Pediatr. (2024) 24(1):747. 10.1186/s12887-024-05225-9 39558280 PMC11572523

[48] Kearney L Kynn M Craswell A Reed R. The relationship between midwife-led group-based versus conventional antenatal care and mode of birth: a matched cohort study. BMC Pregnancy Childbirth. (2017) 17(1):39. 10.1186/s12884-016-1216-1 28103820 PMC5244557

[49] Honorato DJP Fulone I Silva MT Lopes LC. Risks of adverse neonatal outcomes in early adolescent pregnancy using group prenatal care as a strategy for public health policies: a retrospective cohort study in Brazil. Front Public Health. (2021) 9:536342. 10.3389/fpubh.2021.536342 33898367 PMC8062755

[50] Hunter L Da Motta G McCourt C Wiseman O Rayment J Haora P. `It makes sense and it works': maternity care providers' perspectives on the feasibility of a group antenatal care model (pregnancy circles). Midwifery. (2018) 66:56–63. 10.1016/j.midw.2018.07.016 30125782

[51] Lundeen T Musange S Azman H Nzeyimana D Murindahabi N Butrick E. Nurses' and midwives' Experiences of providing group antenatal and postnatal care at 18 health centers in Rwanda: a mixed methods study. PLoS One. (2019) 14(7):e0219471. 10.1371/journal.pone.0219471 31295335 PMC6622527

[52] Jolivet RR Uttekar BV Connor O Lakhwani M Sharma K Wegner J. Exploring perceptions of group antenatal care in urban India: results of a feasibility study. Reprod Health. (2018) 15(1):57. 10.1186/s12978-018-0498-3 29615069 PMC5883286

[53] Wiseman O Emmett L Hickford G Knight M Lazar J Yuill C. The challenges and opportunities for implementing group antenatal care (‘pregnancy Circles') as part of standard NHS maternity care: a co-designed qualitative study. Midwifery. (2022) 109:103333. 10.1016/j.midw.2022.103333 35405404

[54] Lori JR Munro ML Chuey MR. Use of a facilitated discussion model for antenatal care to improve communication. Int J Nurs Stud. (2016) 54:84–94. 10.1016/j.ijnurstu.2015.03.018 25862409 PMC4592694

[55] Lazar J Boned-Rico L Olander EK McCourt C. A systematic review of providers' Experiences of facilitating group antenatal care. Reprod Health. (2021) 18(1):180. 10.1186/s12978-021-01200-0 34493314 PMC8425020

[56] Ahrne M Byrskog U Essén B Andersson E Small R Schytt E. Group antenatal care (gANC) for Somali-speaking women in Sweden—a process evaluation. BMC Pregnancy Childbirth. (2022) 22(1):721. 10.1186/s12884-022-05044-9 36131237 PMC9494829

[57] Ahrne M Schytt E Andersson E Small R Adan A Essén B. Antenatal care for Somali-born women in Sweden: perspectives from mothers, fathers and midwives. Midwifery. (2019) 74:107–15. 10.1016/j.midw.2019.03.022 30953966

[58] Butrick E Lundeen T Phillips BS Tengera O Kambogo A Uwera YDN. Model fidelity of group antenatal and postnatal care: a process analysis of the first implementation of this innovative service model by the preterm birth initiative-Rwanda. Gates Open Res. (2020) 4:7. 10.12688/gatesopenres.13090.1 32363328 PMC7177154

[59] Ibañez-Cuevas M Heredia-Pi IB Fuentes-Rivera E Andrade-Romo Z Alcalde-Rabanal J Cacho LB-B. Group Prenatal Care in Mexico: perspectives and experiences of health personnel. Rev Saude Publica. (2020) 54:140. 10.11606/s1518-8787.2020054002175 PMC7703532 33331532

