# Supplementary material for: Innovation in prenatal care: a global review of implementing group prenatal care and impacts on women, children, and health care providers
Source: Front Glob Womens Health. 2026 Sep 25;7:1910318. doi: 10.3389/fgwh.2026.1910318 (PMC13650468; doi:10.3389/fgwh.2026.1910318)
Supplement: Supplementary file 1 [file Table1.docx]

Supplementary Material

**Table 1. Mini-Review: Description of Articles Included**

| **Reference** | **Location** | **Group Care Setting** | **Duration of Program/ Number of Participant Interactions** | **Study Design** |
| --- | --- | --- | --- | --- |
| ***Impact of group care model of prenatal care on women*** | | | | |
| Adaji et al. (2019) | Nigeria | Rural community ANC program | Avg. 3 visits | Observational study |
| Ahrne et al. (2023) | Sweden | Midwifery-led clinic | 7 sessions | Quasi-experimental (historical control) |
| Casella et al. (2024) | Haiti | J9 Plus program | Pregnancy-postpartum | Mixed-methods study |
| Craswell et al. (2016) | Australia | Collaborative clinic | Not specified | Qualitative evaluation |
| Dai et al. (2022) | China | Internet-based model | Not specified | Qualitative phenomenological study |
| El Aliem et al. (2019) | Egypt | University hospital | 6 sessions | Quasi-experimental study |
| Eluwa et al. (2018) | Nigeria | CenteringPregnancy Groups | Pregnancy-postpartum | Prospective cohort study |
| Ghani (2018) | Egypt | CenteringPregnancy | 6 sessions | Randomized controlled trial |
| Grenier et al. (2019) | Kenya & Nigeria | Health facilities | 5 sessions | Pragmatic cluster RCT |
| Grenier et al. (2022) | Kenya & Nigeria | Group ANC clinics | 5 sessions | Mixed-methods secondary analysis |
| Hawley et al. (2024) | American Samoa | Group prenatal care | Not fully reported | Randomized case control study |
| Hetherington et al. (2018) | Canada | Community health center | Not specified | Cross-sectional survey |
| Hodgson et al. (2017) | Canada | Interprofessional maternity program | Pregnancy | Prospective comparative study |
| Horn et al. (2026) | United States, Australia, Sweden, Canada, United Kingdom, Bangladesh, Kenya, Senegal, The Netherlands, Nepal | Varied | Varied | Systematic review |
| Horn et al. (2025) | Belgium, Ghana, Kosovo, The Netherlands, South Africa, Suriname and The United Kingdom | Varied | Varied | Qualitative meta-thematic analysis |
| Hunter et al. (2019) | England | Pregnancy Circles in community settings | Four circles | Qualitative study |
| Jeremiah et al. (2021) | Malawi & Tanzania | Group ANC+ | Pregnancy | Randomized pilot study |
| Lori et al. (2017) | Ghana | Urban district hospital | Pregnancy | Prospective cohort |
| Lori et al. (2018) | Ghana | Group ANC | Pregnancy–1 year postpartum | Prospective cohort |
| Malchi et al. (2023) | Iran | Group prenatal care | 5 sessions | Randomized controlled trial |
| Marzouk et al. (2018) | Egypt | Antenatal outpatient clinics | Pregnancy | Quasi-experimental study |
| McNeil & Johnston (2016) | Canada | CenteringParenting clinics | 6 visits | Qualitative study |
| Musabyimana et al. (2019) | Rwanda | 18 health centers | ~9 months post-implementation | Qualitative study |
| Patil et al. (2017) | Malawi & Tanzania | Group ANC clinics | Pregnancy | Randomized controlled pilot |
| Patil et al. (2017a) | Malawi & Tanzania | Group ANC clinics | Pregnancy | Randomized controlled pilot |
| Ratzon et al. (2022) | Israel | Community prenatal care | Pregnancy | Semi-randomized community trial |
| Sayed et al. (2018) | Egypt | University hospital clinic | Pregnancy | Quasi-experimental study |
| Somji et al. (2022) | Kenya | 6 health facilities | Pregnancy | Mixed-methods quasi-experimental study |
| Swift et al. (2020) | Australia | Enhanced Antenatal Care model | 6 individual + 4 group visits | Quasi-experimental controlled trial |
| Thapa et al. (2019) | Nepal | Rural community health worker-supported ANC | 1 year | Cluster-controlled effectiveness study |
| Wagijo et al. (2022) | Netherlands | Primary care midwifery centers | Pregnancy | Secondary analysis of stepped-wedge RCT |
| Yang et al. (2023) | China | Tertiary hospital | Prenatal to 42 days postpartum | Randomized controlled trial |
| ***Impact of group care model of prenatal care on infants*** | | | | |
| Akunzirwe et al. (2022) | Uganda | Group vs focused ANC | Pregnancy-postpartum | Cross-sectional comparative study |
| Apetorgbor et al. (2024) | Ghana | 14 health facilities | Pregnancy-12 weeks postpartum | Cluster randomized controlled trial |
| Casella et al. (2024) | Haiti | J9 Plus program | Pregnancy-pediatric follow-up | Mixed-methods study |
| Eluwa et al. (2018) | Nigeria | Centering Pregnancy Groups | Pregnancy-postpartum | Prospective cohort study |
| Hawley et al. (2024) | American Samoa | Group prenatal care | Not fully reported | Randomized case control study |
| Hodgson et al. (2017) | Canada | Interprofessional maternity program | Pregnancy | Prospective comparative study |
| Honorato et al. (2021) | Brazil | Specialized public prenatal services | Pregnancy | Retrospective cohort study |
| Johnston et al. (2017) | Canada | CenteringParenting clinics | First year of life | Pilot study |
| Kearney et al. (2017) | Australia | Midwife-led group care clinic | Pregnancy | Retrospective matched cohort |
| Nicolau et al. (2018) | Spain | Hospital prenatal program | Pregnancy | Retrospective cohort study |
| Noguchi et al. (2020) | Nigeria & Kenya | Group ANC clinics | Pregnancy-postpartum | Secondary analysis of cluster RCT |
| Teasdale et al. (2022) | Kenya | 7 health facilities | Pregnancy-postpartum | Pre-post intervention evaluation |
| Yang et al. (2023) | China | Tertiary hospital | Pregnancy–42 days postpartum | Randomized controlled trial |
| ***Impact of group care model of prenatal care on health care providers*** | | | | |
| Ahrne et al. (2019) | Sweden | ANC services | N/A | Qualitative focus groups |
| Ahrne et al. (2022) | Sweden | Public ANC clinic | 7 sessions | Process evaluation |
| Butrick et al. (2020) | Rwanda | ANC/postnatal clinics | Ongoing | Process analysis |
| Craswell et al. (2016) | Australia | Collaborative clinic | Not specified | Qualitative evaluation |
| Grenier et al. (2022) | Kenya & Nigeria | Group ANC clinics | 5 sessions | Mixed-methods secondary analysis |
| Hunter et al. (2018) | United Kingdom | Pregnancy Circles | Four circles | Qualitative implementation study |
| Ibañez-Cuevas et al. (2020) | Mexico | Four Ministry of Health clinics | 2016–2018 implementation period | Qualitative descriptive study |
| Jolivet et al. (2018) | India | Public and private facilities | N/A | Qualitative feasibility study |
| Lazar et al. (2021) | Nine Country Review | Varied | Varied | Systematic review |
| Lori et al. (2016) | Ghana | Urban district hospital | 3-month pilot | Mixed-methods pilot study |
| Lundeen et al. (2019) | Rwanda | 18 health centers | Approximately 1 year | Mixed-methods study |
| McNeil & Johnston (2016) | Canada | Public Health clinics | 6 visits | Qualitative study |
| Wiseman et al. (2022) | United Kingdom | NHS maternity services | N/A | Qualitative study |
